# Planned early delivery or expectant management for late preterm pre-eclampsia (PHOENIX): a randomised controlled trial

**DOI:** 10.1016/S0140-6736(19)31963-4

**Published:** 2019-09-28

**Authors:** Lucy C Chappell, Peter Brocklehurst, Marcus E Green, Rachael Hunter, Pollyanna Hardy, Edmund Juszczak, Louise Linsell, Virginia Chiocchia, Melanie Greenland, Anna Placzek, John Townend, Neil Marlow, Jane Sandall, Andrew Shennan, PHOENIX Study Group, Umber Agarwal, Irshad Ahmed, Bini Ajay, Zarko Alfirevic, Rita Arya, Gabrielle Bambridge, Jacqueline Bamfo, Sambita Basak, Ursula Bowler, Helen Cameron, David Churchill, Janet Cresswell, Fiona Crosfill, Mark Denbow, Madhuchanda Dey, Caroline Everden, Jo Ficquet, Katarzyna Gajewska-Knapik, Ramesh Ganapathy, Angela Garrett, Joanna Girling, Adam Gornall, Kate Harding, Eleanor Hendy, Richard Howard, Mark James, Antoinette Johnson, Michelle Kemp, Asma Khalil, Rehan Khan, Rahila Khan, Ellen Knox, Lavinia Margarit, Philippa Marsden, Karen McIntyre, Jenny Myers, Justine Nugent, Sanjay Rao, Zoey Robinson, Stephen Robson, Pauline Rushby, Laura Scholz, Mohamed Shahin, Bhavna Sharma, Nigel Simpson, Natasha Singh, Jenie Sparkes, Sophia Stone, Seni Subair, Bee Tan, Vidya Thakur, Sujatha Thamban, Jim Thornton, Jim Thornton, Sue Tohill, Elly Tsoi, Derek Tuffnell, Mark Waterstone, Jason Waugh, Cornelia Wiesender, Pensee Wu

**Affiliations:** a Department of Women and Children's Health, School of Life Course Sciences, King's College London, London, UK; b Birmingham Clinical Trials Unit, University of Birmingham, UK; c Action on Pre-eclampsia, Evesham, UK; d Research Department of Primary Care and Population Health, University College London, London, UK; e UCL EGA Institute for Women's Health, University College London, London, UK; f National Perinatal Epidemiology Unit Clinical Trials Unit, Nuffield Department of Population Health, University of Oxford, Oxford, UK

## Abstract

**Background:**

In women with late preterm pre-eclampsia, the optimal time to initiate delivery is unclear because limitation of maternal disease progression needs to be balanced against infant complications. The aim of this trial was to determine whether planned earlier initiation of delivery reduces maternal adverse outcomes without substantial worsening of neonatal or infant outcomes, compared with expectant management (usual care) in women with late preterm pre-eclampsia.

**Methods:**

In this parallel-group, non-masked, multicentre, randomised controlled trial done in 46 maternity units across England and Wales, we compared planned delivery versus expectant management (usual care) with individual randomisation in women with late preterm pre-eclampsia from 34 to less than 37 weeks' gestation and a singleton or dichorionic diamniotic twin pregnancy. The co-primary maternal outcome was a composite of maternal morbidity or recorded systolic blood pressure of at least 160 mm Hg with a superiority hypothesis. The co-primary perinatal outcome was a composite of perinatal deaths or neonatal unit admission up to infant hospital discharge with a non-inferiority hypothesis (non-inferiority margin of 10% difference in incidence). Analyses were by intention to treat, together with a per-protocol analysis for the perinatal outcome. The trial was prospectively registered with the ISRCTN registry, ISRCTN01879376. The trial is closed to recruitment but follow-up is ongoing.

**Findings:**

Between Sept 29, 2014, and Dec 10, 2018, 901 women were recruited. 450 women (448 women and 471 infants analysed) were allocated to planned delivery and 451 women (451 women and 475 infants analysed) to expectant management. The incidence of the co-primary maternal outcome was significantly lower in the planned delivery group (289 [65%] women) compared with the expectant management group (338 [75%] women; adjusted relative risk 0·86, 95% CI 0·79–0·94; p=0·0005). The incidence of the co-primary perinatal outcome by intention to treat was significantly higher in the planned delivery group (196 [42%] infants) compared with the expectant management group (159 [34%] infants; 1·26, 1·08–1·47; p=0·0034). The results from the per-protocol analysis were similar. There were nine serious adverse events in the planned delivery group and 12 in the expectant management group.

**Interpretation:**

There is strong evidence to suggest that planned delivery reduces maternal morbidity and severe hypertension compared with expectant management, with more neonatal unit admissions related to prematurity but no indicators of greater neonatal morbidity. This trade-off should be discussed with women with late preterm pre-eclampsia to allow shared decision making on timing of delivery.

**Funding:**

National Institute for Health Research Health Technology Assessment Programme.

## Introduction

Pre-eclampsia is a multisystem disorder of pregnancy, characterised by placental and maternal vascular dysfunction and associated with substantial morbidity and mortality for the mother and infant. Adverse outcomes of pre-eclampsia include maternal stroke, renal and hepatic injury, fetal growth restriction, and maternal and perinatal death.^1^ Around 10% of pregnant women develop hypertension and 2–3% develop pre-eclampsia, characterised by hypertension and manifestations of multiorgan disease.^2^

Standard management of pre-eclampsia involves maternal and fetal assessment and subsequent consideration of timely delivery to minimise maternal and perinatal morbidity, taking into consideration gestational age, progression of maternal disease, and fetal wellbeing. After 37 weeks' gestation, most national guidelines recommend prompt delivery for a woman with pre-eclampsia^3^, ^4^ because maternal risks can be significantly reduced without additional perinatal risks from such an intervention.^5^ In women with late preterm pre-eclampsia (between 34 and 37 weeks' gestation), the optimal time for delivery is less clear, because limitation of maternal disease progression needs to be balanced against complications for the infant either related to ongoing expectant management (including needing emergency delivery, worsening growth restriction, and stillbirth) or those related to planned earlier delivery (infant immaturity and associated complications). Current usual practice in the UK for women with late preterm pre-eclampsia is for expectant management until 37 weeks' gestation, with delivery sooner if the clinical scenario changes and there is concern over impending severe pre-eclampsia and associated complications. In the absence of definitive new evidence, this advice has been maintained in the most recent management recommendations from the International Society for the Study of Hypertension in Pregnancy, published in 2018,^6^ which are used to inform current practice in many countries worldwide.

**Research in context**

**Evidence before this study**

At conception of this trial in June, 2012, we searched PubMed for studies in humans published in any language using the following MEDLINE subject heading keywords: “gestational age”; “hypertension, pregnancy-induced”; “labor, induced”; “obstetric delivery”; “pre-eclampsia”; “pregnancy”; and “pregnancy outcome”. We found no published randomised controlled trials evaluating planned delivery against expectant management for women with late preterm pre-eclampsia between 34 and 37 weeks' gestation, although some women with mild pre-eclampsia might have been included in the HYPITAT-1 trial, in women with pregnancy hypertension from 36 weeks' gestation. A Cochrane systematic review that assessed timing of delivery interventions for women with hypertensive disorders of pregnancy from 34 weeks' gestation to term, updated on Jan 15, 2017, included five studies, all published after 2012 with the exception of one large trial (published in 2009) that included women with pregnancy hypertension from 36 weeks' gestation onwards (and therefore only indirectly relevant to the research uncertainty assessed here). Taking all the studies into account, the Cochrane systematic review concluded that planned early delivery was associated with fewer maternal complications but no clear differences in infant outcomes. However, they cautioned that few data were available, particularly on infant outcomes, from trials where women with all hypertensive disorders (gestational hypertension, chronic hypertension, and pre-eclampsia) were considered as one group. They advised that further studies were needed to look at optimal timing of delivery, particularly in different types of pregnancy hypertensive disorders.

**Added value of this study**

This large, multicentre trial represents contemporaneous management of women with late preterm pre-eclampsia. Our sample size (901 women) is considerably larger than the number of women with late preterm pre-eclampsia in previous trials included in the Cochrane systematic review (352 and 183 women) that considered the same gestational age window. Those trials did not affect clinical practice as there was continued uncertainty over the trade-off between maternal benefit and perinatal harms. The neonatal endpoint chosen in our trial reflects potential harms from both the intervention (planned early delivery) and ongoing pre-eclampsia (in the expectant management group).

**Implications of all the available evidence**

The results of this trial, taken together with smaller trials published since the trial started, support a lower threshold for considering planned delivery in women with late preterm pre-eclampsia. This benefit seems to be greater in women with pre-eclampsia (compared with women in other studies with gestational or chronic hypertension alone). Although planned delivery might result in more infants being admitted to a neonatal unit under current guidelines, the observed lack of associated morbidity and provision of alternative care strategies that avoid separation of the infant from their mother (such as transitional care) should enable management of these women to be optimised.

The aim of this trial was to compare planned earlier initiation of delivery versus expectant management (usual care) in women with pre-eclampsia between 34 and 37 weeks' gestation in the UK to determine whether planned delivery reduces maternal adverse outcomes without substantial worsening of neonatal or infant outcomes.

## Methods

### Study design and participants

In this parallel-group, non-masked, multicentre, randomised controlled trial, we compared planned delivery against expectant management (usual care). The trial was done in 46 consultant-led maternity units in England and Wales.

A pregnant woman was eligible if she was between 34 weeks and less than 37 weeks of gestation, had a diagnosis of pre-eclampsia or superimposed pre-eclampsia (as defined by the International Society for the Study of Hypertension in Pregnancy),^7^ with a singleton or dichorionic diamniotic twin pregnancy and at least one viable fetus, was aged 18 years or older, and was able to give written informed consent. Women with any other comorbidity (including pre-existing hypertension or diabetes) or with a previous caesarean section or any fetal position were eligible. The only exclusion criterion to study participation was if a decision had already been made to deliver within the next 48 h. Current practice by national guidelines in use during the trial was for immediate delivery of a woman with persistent severe features of pre-eclampsia (including haemolysis, elevated liver enzymes, and low platelets syndrome); these women would thus not be eligible for the trial.

The trial protocol has been previously published.^8^ There were no substantial changes to the published study design, methods, or outcomes after the start of the trial. The trial was approved by the South Central—Hampshire B Research Ethics Committee (no 13/SC/0645).

### Randomisation and masking

Participants were randomly assigned to planned delivery or expectant care in a 1:1 ratio using a probabilistic minimisation algorithm to ensure approximate balance within the following groups: study centre, singleton or twin pregnancy, severity of hypertension in 48 h before enrolment (highest systolic blood pressure with or without medication: <150 mm Hg, 150–159 mm Hg, or ≥160 mm Hg), parity (previous delivery of an infant past 24 weeks' gestation), previous caesarean section, and gestational age at randomisation (34, 35, or 36 weeks). Randomisation was managed via a secure web-based randomisation program provided by MedSciNet. The minimisation algorithm was implemented by a MedSciNet database programmer, with balance and predictability monitored by the independent National Perinatal Epidemiology Unit Clinical Trials Unit statistician during the trial. The intervention was not masked from women, clinicians, or data collectors due to the nature of the intervention. Trial statisticans were also not blinded to allocation.

### Procedures

We allocated women to planned initiation of delivery within 48 h of randomisation (to allow for corticosteroid administration to accelerate fetal lung maturation and neonatal cot availability if necessary) or to expectant management (usual care). Planned delivery was usually by induction of labour, unless there was an additional specific indication for pre-labour caesarean section. Expectant management involved delivery at 37 weeks' gestation or sooner as clinical needs dictated in accordance with the UK national guidelines,^4^ as assessed by the clinician responsible for the woman's care, for maternal indications (eg, uncontrolled hypertension or abnormal blood results), fetal compromise, eclampsia, or other clinical crises. Individual decisions around mode of induction and delivery and use of corticosteroids for fetal lung maturity were left to the discretion of the individual clinician, with the trial protocol advising that all options should be discussed with the pregnant woman and her needs and preferences taken into account.

Site research teams approached women to confirm eligibility and provided verbal and written information. A trained clinician (obstetrician or obstetric physician) obtained written informed consent. A research team member entered baseline data on a web-based database and then performed randomisation, communicating the results directly to the woman. All other aspects of pregnancy management were expected to be in accordance with the UK national guidelines^4^ at the discretion of the responsible clinician.

Outcomes were recorded on the web-based trial database through case-note review by trained researchers after maternal and infant primary hospital discharge. Participants were asked to complete the EuroQol five dimensions, five levels questionnaire (EQ-5D-5L) at baseline to assess health-related quality of life. Planned long-term follow-up assessments include the EQ-5D-5L, 12-item Short Form Health Survey, and maternal and infant health-care and social care use after hospital discharge at 6 months post-delivery and when infants are 2 years of age, corrected for prematurity. In addition, the Parent Report of Children's Abilities—Revised (PARCA-R) questionnaire will be collected at 2 years.

### Outcomes

The co-primary short-term maternal outcome was a composite of maternal morbidity of fullPIERS^9^ outcomes, with the addition of recorded systolic blood pressure of at least 160 mm Hg post randomisation (on any occasion). fullPIERS outcomes were maternal death; central nervous system (eclampsia, Glasgow coma score <13, stroke or reversible ischaemic neurological deficit, transient ischa-emic attack, cortical blindness or retinal detachment, or posterior reversible encephalopathy); cardiorespiratory (positive inotropic support, infusion of a third parenteral antihypertensive drug, myocardial ischaemia or infarction, peripheral oxygen saturation <90%, ≥50% fraction of inspired oxygen for >1 h, intubation [other than for caesarean section], or pulmonary oedema); haematological (transfusion of any blood product or platelet count <50 × 10^9^ per L with no transfusion); hepatic (hepatic dysfunction or hepatic haematoma or rupture); renal (acute renal insufficiency [creatinine >150 μmol/L with no pre-existing renal disease], acute renal failure [creatinine >200 μmol/L with pre-existing renal disease], or dialysis); or placental abruption. Presence or absence of the co-primary maternal outcome was independently countersigned by the site principal investigator or delegate.

The co-primary short-term perinatal outcome was a composite of neonatal deaths within 7 days of delivery and perinatal deaths or neonatal unit admissions (physical separation of an infant from their mother) before infant hospital discharge. The primary long-term infant outcome will be the PARCA-R composite score for neurodevelopment at 2 years of age, corrected for prematurity, and will be assessed when data collection has been completed.

Secondary outcomes are as listed in the published protocol.^8^ Maternal outcomes comprised individual components of the composite primary outcome (maternal morbidity of fullPIERS outcomes or recorded systolic blood pressure of at least 160 mm Hg); use of antihypertensive drugs; progression to severe pre-eclampsia, defined as systolic blood pressure of at least 160 mm Hg, platelet count less than 100 × 10^9^ per L, and abnormal liver function enzymes (alanine aminotransferase or aspartate aminotransferase >70 IU/L); time and mode of onset; confirmed thromboembolic disease; confirmed sepsis; primary and additional indications for delivery; and placental abruption. Perinatal outcomes comprised stillbirth, neonatal death within 7 days of delivery, neonatal death before hospital discharge, admissions to neonatal unit, number of nights in each category of care, total number of nights in hospital, birthweight, birthweight centile, birthweight less than tenth or third centile, gestational age at delivery, Apgar score at 5 min after birth, umbilical arterial and venous pH at birth, need for supplementary oxygen before discharge, number of days when supplemental oxygen is required, need for respiratory support, other indications and main diagnoses resulting in neonatal unit admission, and health resource use outcomes. The primary indication for neonatal unit admission was allocated as part of usual clinical care practice by a clinical neonatologist (not involved in the trial), from a prespecified list of exclusive admission reasons, on an electronic clinical database used nationwide in England and Wales. The category of neonatal care (intensive care, high-dependency care, or special care) followed nationally defined guidance, with days in each category of care individually recorded on the national electronic clinical patient database.^10^

Short-term health economic and quality-of-life outcomes were number of maternal hospital attendances and nights, cost of delivery, and cost of neonatal care.

Long-term health economic and quality-of-life outcomes to be assessed when data collection has been completed include quality of maternal physical and mental health when the infant is 6 months and 2 years old (corrected for prematurity), quality of life as assessed by the EQ-5D-5L questionnaire, retrospective 6-month health-care and social care use by mother and infant at 6 months and 2 years, and maternal quality-adjusted life-years at 2 years.

Research teams undertook standard assessments of safety, with reporting of adverse events and serious adverse events following usual governance procedures for a clinical trial.

### Statistical analysis

Assuming an anticipated composite adverse maternal outcome incidence of 43% in the expectant management group, based on data from the PELICAN study,^11^ a sample size of 850 women would show a relative risk reduction of 25% (from 43·00% to 32·25%; deemed clinically important) in the planned delivery group with a two-sided 5% significance level and 90% power. With 5% loss of women in follow-up, the overall target for recruitment was 900 women (450 per group).

Assuming a composite adverse neonatal outcome incidence of 24% in the expectant management group^11^ and assuming a sample size of 850 women would result in approximately 860 infants (430 per group, allowing for twin births), 93% power would be achieved to detect a non-inferiority margin of no less than 10% (judged as clinically relevant) and 78% power to detect a margin of no less than 8%.

The primary analysis for all maternal outcomes was by intention to treat with participants analysed in the groups to which they were assigned regardless of protocol non-compliances. The primary analysis for all perinatal and infant outcomes was by both intention to treat and per protocol, since the hypothesis under examination for these outcomes was a non-inferiority hypothesis.

All outcomes were analysed adjusting for minimisation factors at randomisation.^12^ Binary outcomes were analysed using mixed-effect Poisson regression with a robust variance estimator and presented as adjusted relative risk (RR) with associated 95% CIs. Site was treated as a random effect and all other minimisation factors as fixed effects. For perinatal outcomes, mothers' identification was nested within site to take account of clustering within twins. For continuous outcomes, differences in medians and associated 95% CIs were estimated using quantile regression. In these models, site was treated as a fixed effect and robust SEs were used. 95% CIs are presented for all primary and secondary outcomes. No adjustment for multiplicity was made for the co-primary outcomes.^13^

Prespecified subgroup analyses were done for co-primary outcomes, using the statistical test of interaction, based on criteria selected for minimisation: parity (no previous pregnancies *vs* ≥1 previous pregnancy), highest systolic blood pressure in the 48 h before enrolment (<150 mm Hg *vs* ≥150 mm Hg), gestation at the time of randomisation (34 weeks *vs* 35 weeks *vs* 36 weeks) and singleton versus twin pregnancy. To allow for clinical and logistical delays, we did a prespecified sensitivity analysis on the co-primary outcomes excluding women and infants randomised to the planned delivery group where initiation of delivery was more than 96 h post randomisation.

Data on mother and infant inpatient care and mode of delivery were costed using the National Schedule of Reference costs.^14^ Descriptive statistics are reported, including mean cost per participant and 95% CIs constructed using bootstrapping with 7000 bootstrap replications. The time horizon of the analysis is from recruitment until hospital discharge following labour. The comparative difference in costs was calculated using linear regressions and adjusted for centre, singleton or twin pregnancies, severity of hypertension in the 48 h before enrolment, parity, previous caesarean section, and gestational age at randomisation.

Data analyses were done with STATA/SE version 15.1. The trial is registered with the ISRCTN registry, ISRCTN01879376.

### Role of the funding source

The funder of the study had no role in study design, data collection, data analysis, data interpretation, or writing of the report. The corresponding author had full access to all the data in the study and had final responsibility for the decision to submit for publication.

## Results

**Figure 1:**
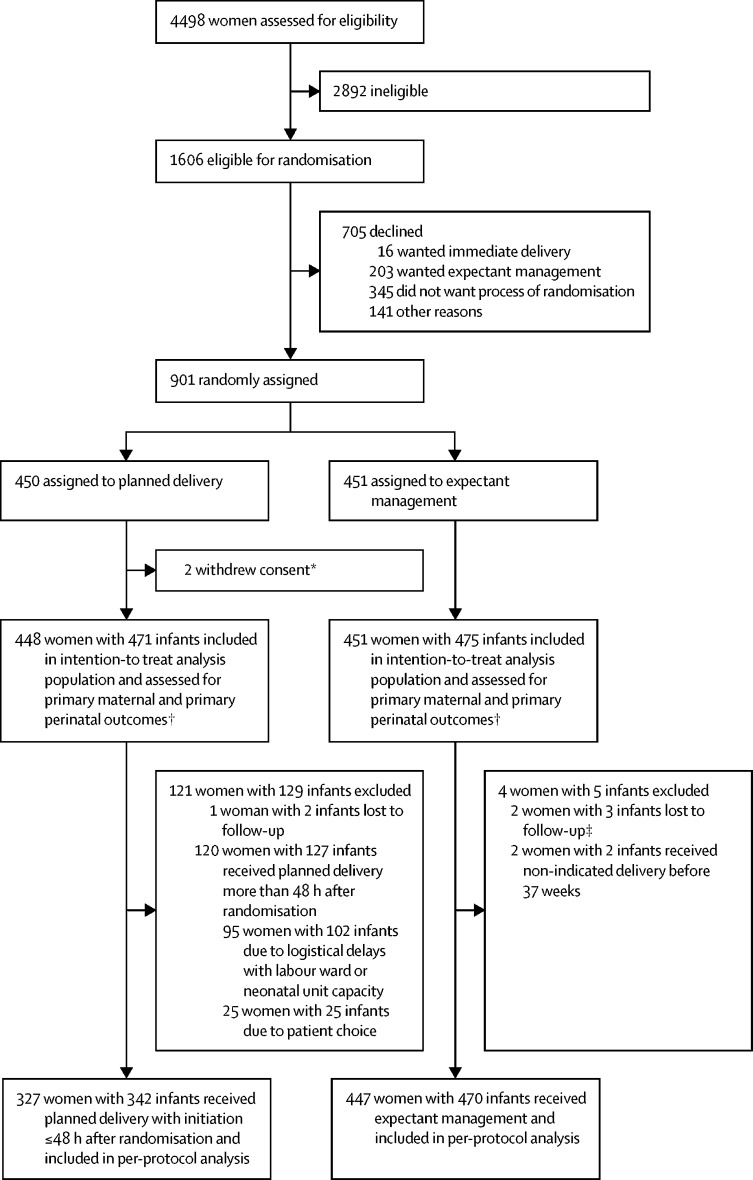
Trial profile *These women withdrew from the trial and withdrew consent for data already collected to be used so are excluded from all analyses. One withdrew before initiation of delivery, the other withdrew after receiving planned delivery within 48 h. †Includes all infants of all women included in the primary maternal outcome. ‡One woman in this group had documented delivery before 37 weeks (on electronic health records) but no further information available.

Between Sept 29, 2014, and Dec 10, 2018, 1606 women were found to be eligible, of whom 901 (56%) were recruited, across 46 maternity units in England and Wales (appendix pp 2–3). 450 women were allocated to planned delivery and 451 women to expectant management (figure 1). For the intention-to-treat analysis, data from 448 women and 471 infants in the planned delivery group and 451 women and 475 infants in the expectant management group were included. Follow-up to maternal and infant discharge continued until Dec 28, 2018. One woman was lost to follow-up in the planned delivery group and two women in the expectant management group (figure 1).

Baseline characteristics seemed similar between the two groups, with groups well balanced on minimisation factors (table 1; appendix pp 4–7).

**Table 1:** Maternal demographic and pregnancy characteristics at baseline and randomisation

		**Planned delivery (n=448)**	**Expectant management (n=451)**
**Baseline characteristics**			
Maternal age, years		30·6 (6·4)	30·8 (6·3)
Ethnicity			
	White	313 (70%)	311 (69%)
	Mixed	10 (2%)	23 (5%)
	Asian	60 (13%)	50 (11%)
	Chinese	0	1 (<1%)
	Black	58 (13%)	52 (12%)
	Other	5 (1%)	13 (3%)
	Unknown	2 (<1%)	1 (<1%)
Deprivation index quintile 5 (most deprived)*		161/425 (38%)	160/428 (37%)
Parity†			
	No previous births	254 (57%)	260 (58%)
	≥1 previous birth	194 (43%)	191 (42%)
Previous caesarean section†		77/194 (40%)	78/191 (41%)
History of pre-eclampsia		85/194 (44%)	92/191 (48%)
Body-mass index at booking, kg/m^2^		29·8 (7·3)	29·8 (7·2)
Smoking at booking		53 (12%)	50 (11%)
Systolic blood pressure at booking, mm Hg		118·7 (14·4)	119·6 (13·7)
Diastolic blood pressure at booking, mm Hg		72·7 (10·2)	73·4 (10·4)
Pre-existing chronic hypertension		51 (11%)	53 (12%)
Pre-existing chronic renal disease		6 (1%)	4 (1%)
Pre-pregnancy diabetes		25 (6%)	28 (6%)
Gestational diabetes		62 (14%)	53 (12%)
Aspirin prescribed during pregnancy		170 (38%)	189 (42%)
LMWH prescribed during pregnancy		125 (28%)	117 (26%)
**Characteristics at randomisation**			
Median gestational age, weeks		35·6 (34·7–36·3)	35·6 (34·7–36·3)
Gestational age category†			
	34 to <35 weeks	131 (29%)	135 (30%)
	35 to <36 weeks	137 (31%)	132 (29%)
	36 to <37 weeks	180 (40%)	184 (41%)
Number of live fetuses†			
	Singleton	425 (95%)	427 (95%)
	Dichorionic diamniotic twin	23 (5%)	24 (5%)
Highest systolic blood pressure in previous 48 h, mm Hg		154·5 (14·5)	155·2 (15·4)
Highest diastolic blood pressure in previous 48 h, mm Hg		95·7 (9·5)	95·8 (10·1)
Highest blood pressure in previous 48 h†			
	≤149 mm Hg	163 (36%)	163 (36%)
	150–159 mm Hg	121 (27%)	123 (27%)
	≥160 mm Hg	164 (37%)	165 (37%)
Urinary protein–creatinine ratio measured		434 (97%)	441 (98%)
Urinary protein–creatinine ratio, mg/mmol		83 (42–186)	80 (42–172)
Fetal growth ultrasound in previous 2 weeks		366 (82%)	375 (83%)
	Suspected fetal growth restriction on ultrasound	79/366 (22%)	85/375 (23%)
Cervical assessment (before randomisation)			
	Bishop's score <2	2 (<1)	2 (<1%)
	Bishop's score 2–6	7 (2%)	4 (1%)
	Not assessed	439 (98%)	445 (99%)
Inpatient at time of randomisation		362 (81%)	371 (82%)

Data are n (%), mean (SD), or median (IQR). LMWH=low molecular weight heparin.

* Deprivation quintiles calculated for participants in England only (not available for participants in Wales).

† Minimisation factors used to ensure balance at randomisation.

The proportion of women with the primary maternal outcome was significantly lower in the planned delivery group compared with the expectant management group (adjusted RR 0·86, 95% CI 0·79–0·94; p=0·0005; table 2). The proportion of infants with the primary perinatal outcome was significantly higher in the planned delivery group compared with the expectant management group (1·26, 1·08–1·47; p=0·0034), with similar results in the per-protocol analysis (table 2). The 95% CIs for the risk difference, for both the intention-to-treat and per-protocol analysis, exclude zero and contain the non-inferiority margin of 10%; hence, we can conclude that planned delivery is inferior to expected management in regard to the primary perinatal outcome.

**Table 2:** Primary maternal and perinatal outcomes

		**Planned delivery**	**Expectant management**	**Effect measure**	Adjusted effect measure*
**Maternal co-primary outcome**					
Intention-to-treat analysis		289/448 (65%)	338/451 (75%)	RR 0·86 (0·79–0·94); p=0·0006	RR 0·86 (0·79–0·94); p=0·0005
**Perinatal co-primary outcome**					
Intention-to-treat analysis		196/471 (42%)	159/475 (34%)	RR 1·25 (1·05–1·48); p=0·0107	RR 1·26 (1·08–1·47); p=0·0034
	Risk difference	..	..	0·08 (0·02–0·15)	0·07 (0·02–0·13)
Per-protocol analysis		155/342 (45%)	155/470 (33%)	RR 1·37 (1·15–1·64); p=0·0005	RR 1·40 (1·18–1·66); p<0·0001
	Risk difference	..	..	0·12 (0·05–0·19)	0·11 (0·05–0·17)

Data are n/N (%), RR (95% CI); p value, or risk difference (95% CI) for the non-inferiority analysis. RR=relative risk.

* Adjusted for centre, singleton or twin pregnancies, severity of hypertension in 48 h before enrolment, parity, previous caesarean section, and gestational age at randomisation.

A significant reduction in both components of the primary adverse maternal outcome was found in women assigned to planned delivery compared with expectant management, as was progression to severe pre-eclampsia (table 3). Other than two women who had spontaneous onset of labour, all other women in the planned delivery group received the trial intervention, although this was not always initiated within 48 h as intended. Of women allocated to planned delivery, 327 (73%) of 448 had delivery initiated within 48 h (figure 1). In women allocated to expectant management, 244 (54%) of 451 women had medically indicated delivery before 37 weeks' gestation and only two women gave birth before 37 weeks' gestation without an additional medical indication. Additional maternal secondary outcomes are shown in the appendix (pp 8–10) along with intervals between randomisation and initiation of delivery (appendix p 11).

**Table 3:** Secondary maternal outcomes post-randomisation

		**Planned delivery (n=448)**	**Expectant management (n=451)**	Adjusted relative risk*(95% CI)
Maternal morbidity composite outcome		68 (15%)	90 (20%)	0·76 (0·59–0·98)
Systolic blood pressure ≥160 mm Hg		267 (60%)	313 (69%)	0·85 (0·77–0·94)
Progression to severe pre-eclampsia		287 (64%)	334 (74%)	0·86 (0·79–0·94)
Placental abruption		4 (1%)	4 (1%)	1·00 (0·37–2·67)
Antihypertensive medication before delivery		381 (85%)	405 (90%)	0·95 (0·91–0·99)
Onset of labour				
	Spontaneous	2 (<1%)	19 (4%)	0·11 (0·02–0·50)
	Induced	304 (68%)	275 (61%)	1·11 (1·01–1·23)
	Pre-labour caesarean section	140 (31%)	152 (34%)	0·93 (0·76–1·13)
	PROM and augmentation	1 (<1%)	4 (1%)	..
Indication for delivery (non-exclusive)†				
	Spontaneous labour <37 weeks' gestation	2 (<1%)	19 (4%)	..
	Trial allocation to planned delivery arm	445 (99%)	0	..
	Reaching 37 weeks' gestation	8 (2%)	188 (42%)	..
	Uncontrolled maternal hypertension	26 (6%)	111 (25%)	..
	Maternal haematological abnormality	3 (1%)	23 (5%)	..
	Maternal biochemical abnormality	19 (4%)	57 (13%)	..
	Fetal compromise on ultrasound scan	16 (4%)	50 (11%)	..
	Fetal compromise on cardiotocography	33 (7%)	64 (14%)	..
	Severe maternal symptoms	9 (2%)	48 (11%)	..
	Other (with none of the above)	0 (<1%)	2 (<1%)	..
Maternal complications before discharge				
	Confirmed thromboembolic disease	0	0	..
	Confirmed sepsis (positive blood or urine cultures)	2 (<1%)	6 (1%)	0·36 (0·07–1·74)

Relative risks are shown for prespecified analyses only. PROM=prelabour rupture of membranes.

* Adjusted for centre, singleton or twin pregnancies, severity of hypertension in 48 h before enrolment, parity, previous caesarean section, and gestational age at randomisation.

† Indications for delivery were predefined in the protocol.

Median gestational age at enrolment was identical in both treatment groups (table 1), but women allocated to planned delivery gave birth at 252 days of gestation compared with 257 days in the expectant management group and were significantly more likely to achieve a spontaneous vaginal delivery (table 4). There were no stillbirths or neonatal deaths in either group. More infants were admitted to the neonatal unit in the planned delivery group than in the expectant management group; the principal recorded indication for admission was prematurity (table 4). There were no differences in the proportions requiring supplementary oxygen or additional respiratory support, legth of stay in different categories of neonatal care, or overall length of stay for the infant (table 4). Additional perinatal secondary outcomes are shown in the appendix (pp 12–14). A prespecified per-protocol analysis gave similar results to the intention-to-treat analysis for perinatal outcomes (appendix pp 15–19).

**Table 4:** Secondary perinatal outcomes by intention to treat

			**Planned delivery (n=471)**	**Expectant management (n=475)**	Adjusted effect measure*(95% CI)
Stillbirth			0	0	..
Neonatal death within 7 days of delivery			0	0	..
Neonatal death before discharge			0	0	..
Median gestational age at delivery, days			252 (246 to 257)	257 (251 to 260)	−3·0 (−3·5 to −2·5)
Mode of delivery					
	Spontaneous vaginal		169 (36%)	139 (29%)	1·21 (1·04 to 1·41)
	Assisted vaginal		40 (9%)	47 (10%)	0·87 (0·61 to 1·26)
	Caesarean section		260 (55%)	289 (61%)	0·92 (0·84 to 1·01)
Median birthweight, g			2405 (2070 to 2753)	2480 (2150 to 2910)	−85 (−137 to −33)
Median birthweight centile†			35 (17 to 61)	30 (13 to 61)	4·2 (−0·4 to 8·7)
Birthweight less than tenth centile			74 (16%)	95 (20%)	0·79 (0·58 to 1·09)
Birthweight less than third centile			20 (4%)	27 (6%)	0·77 (0·43 to 1·38)
Apgar score at 5 min after birth			10 (9 to 10)	10 (9 to 10)	0·0 (−0·3 to 0·3)‡
Median umbilical arterial pH			7·26 (7·20 to 7·30)	7·25 (7·20 to 7·30)	0·00 (−0·01 to 0·01)
Umbilical arterial pH collected			281 (60%)	266 (56%)	..
Infants admitted to neonatal unit			196 (42%)	159 (34%)	1·26 (1·08 to 1·47)
Principal recorded indication for neonatal unit admission§					
	Prematurity		83/196 (42%)	40/159 (25%)	..
	Respiratory disease		47/196 (24%)	41/159 (26%)	..
	Hypoglycaemia		21/196 (11%)	31/159 (20%)	..
	Jaundice		12/196 (6%)	11/159 (7%)	..
	Infection suspected or confirmed		9/196 (5%)	12/159 (8%)	..
	Intrauterine growth restriction or infant small for gestational age		8/196 (4%)	10/159 (6%)	..
	Other		16/196 (8%)	14/159 (9%)	..
Need for respiratory support			45 (10%)	48 (10%)	0·97 (0·60 to 1·57)
Need for supplementary oxygen before discharge			60 (13%)	49 (10%)	1·26 (0·89 to 1·79)
Days of supplemental oxygen required			1 (1 to 2)	2 (1 to 3)	..
Total time in neonatal unit					
	Days		6 (3 to 11)	6 (3 to 12)	0·0 (−1·3 to 1·3)
	Number admitted for at least 1 day		181 (39%)	153 (33%)	..
Category of care during neonatal unit stay (separation of baby from mother)					
	Time in intensive care				
		Days	2 (1 to 3)	3 (1 to 4)	−1·3 (−18·3 to 15·6)
		Number admitted	27 (6%)	19 (4%)	..
	Time in high-dependency care				
		Days	2 (1 to 3)	2 (1 to 4)	−0·5 (−1·5 to 0·5)
		Number admitted	51 (11%)	33 (7%)	..
	Time in special care				
		Days	6 (2 to 10)	6 (2 to 11)	0·0 (−1·4 to 1·4)
		Number admitted	168 (36%)	143 (31%)	..
Category of care during other postnatal stay (baby alongside mother)					
	Time in transitional care				
		Days	5 (2 to 8)	5 (4 to 6)	0·50 (−14·38 to 15·38)
		Number admitted	40 (9%)	16 (3%)	..
	Time in postnatal care				
		Days	3 (2 to 5)	3 (2 to 4)	0·50 (0·28 to 0·72)
		Number admitted	350 (75%)	384 (82%)	..

Data are n (%), n/N (%), or median (IQR). Effect measures are relative risks for categorical variables (risk in planned delivery group : risk in expectant management group) and median differences for continuous variables (median in planned delivery group – median in expectant management group), and are given for prespecified analyses only.

* Adjusted for centre, singleton or twin pregnancies, severity of hypertension in 48 h before enrolment, parity, previous caesarean section, and gestational age at randomisation.

† Birthweight centile calculated using the Stata add-in function zanthro using the British 1990 Growth Reference (reanalysed 2009).

‡ Unadjusted effect measures (adjusted measure could not be calculated).

§ Full list of other indications for neonatal unit admission given in the appendix (pp 12–14).

Total maternal and infant costs were lower in the planned delivery group compared with the expectant management group, with an adjusted cost saving of £1478 (95% CI 2354–605; p=0·00094; table 5).

**Table 5:** Health economic evaluation of costs

		**Planned delivery (448 women, 471 infants)**	**Expectant management (451 women, 475 infants)**
Maternal costs			
	Antenatal inpatient	£1261 (1120 to 1401)	£2892 (2619 to 3164)
	Labour and delivery	£6087 (5750 to 6425)	£5468 (5107 to 5830)
	Maternal intensive therapy and high-dependency units	£422 (314 to 530)	£610 (474 to 746)
	Maternal outpatient	£68 (45 to 91)	£292 (238 to 345)
	Maternal transfer	£30 (−12 to 71)	£65 (−37 to 168)
	Total	£8238 (7848 to 8628)	£9866 (9342 to 10 392)
Infant costs			
	Infant intensive care	£198 (95 to 301)	£362 (3 to 721)
	Infant high-dependency care	£239 (154 to 324)	£203 (106 to 300)
	Infant special care	£1402 (1152 to 1653)	£1257 (1013 to 1500)
	Infant normal and transitional care	£1515 (1379 to 1651)	£1401 (1274 to 1529)
	Total	£3354 (3048 to 3661)	£3223 (2763 to 3684)
Total maternal and infant costs		£11 574 (10 981 to 12 167)	£13 090 (12 326 to 13 855)

Data are mean (95% CI).

There were similar numbers of serious adverse events in both groups: nine in the planned delivery group compared with 12 in the expectant management group (appendix p 20). Two serious adverse events in each group were judged possibly related to the intervention in each group; one serious adverse event was judged probably related to the intervention in the expectant management group. All other serious adverse events were deemed unrelated to the intervention. There was one maternal death in the expectant management group, in a woman with underlying medical comorbidities who collapsed unexpectedly 5 days after delivery; this death was considered unrelated to the trial allocation.

**Figure 2:**
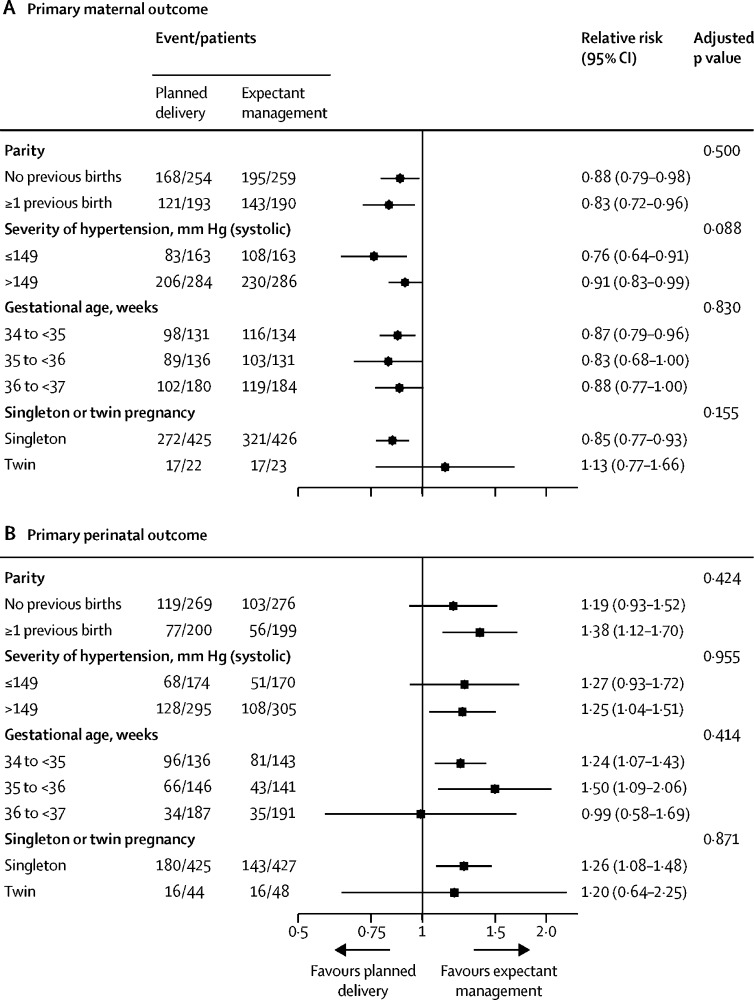
Forest plot for subgroup analysis comparing planned delivery with expectant management Forest plots show analysis of primary maternal outcome (A) and primary perinatal outcome (B). Analysis is by intention to treat. p values compare relative risks across the different subgroups of each factor.

In prespecified subgroup analyses, we found no significant interaction between the incidence of the primary maternal or perinatal outcome and gestational age at randomisation, singleton or twin pregnancy, highest systolic blood pressure before enrolment, or parity (figure 2; appendix p 21). A prespecified sensitivity analysis excluding women or infants randomly assigned to the planned delivery group with initiation of delivery after 96 h had little effect on the results (appendix p 22).

## Discussion

In this randomised controlled trial in women with late preterm pre-eclampsia, planned delivery reduced maternal morbidity, including severe systolic hypertension. However, planned delivery led to more neonatal unit admissions for the infant, principally for a listed indication of prematurity and without an excess of respiratory or other morbidity, intensity of care, or length of stay. Women in the expectant management group had a median additional prolongation of pregnancy from enrolment to delivery of 5 days (3 days after adjustment) and more than half of these women had indicated delivery before 37 weeks' gestation, with three quarters subsequently meeting the criteria for progression to severe pre-eclampsia. Women in the planned delivery group had significantly more spontaneous vaginal deliveries. In this health-care setting, there were no stillbirths or neonatal deaths. We found that planned delivery had lower costs than expectant management in this setting.

Strengths of the trial include a sufficiently large sample of women specifically with late preterm pre-eclampsia, in whom the benefits and risks of planned delivery might be different from those with gestational or chronic hypertension in pregnancy, related to the likelihood of progression to severe features of the disease and need for medically indicated emergency delivery. The trial was conducted to rigorous standards, with a prespecified protocol without changes. Findings are likely to be generalisable to similar health-care settings, because it was undertaken in a large number of maternity units across England and Wales, with diverse representation of women in terms of both demography and disease spectrum. Recommendations for expectant management and indications for delivery in our trial followed UK national guidelines^4^ and current international guidelines,^6^ so our findings are relevant to countries that have similar recommendations. More than half of eligible women approached agreed to participate in the trial, indicating agreement of equipoise in this scenario.

Limitations of the trial include the challenge of finding a perinatal outcome that adequately represented the potential risks of both groups—related to intervention in the planned delivery group and to ongoing pre-eclampsia in the expectant management group—as there are potential harms from continuing pregnancy as well as initiating earlier delivery. Because adjudication of multiorgan neonatal morbidity is complex and subjective, and no widely accepted validated measure of neonatal morbidity is currently available, we chose neonatal unit admission (involving separation of the infant from their mother), supported by our lay representatives, and intending that this would capture underlying neonatal morbidity. Although UK clinical practice guidelines do not recommend routine admission of an infant based solely on gestational age after 34 weeks of pregnancy, admission principally for prematurity in this trial suggests different real-world clinician behaviour, despite no differences in objective measures of direct neonatal morbidity being shown. Choice of a maternal outcome that reflects the multiorgan manifestations of pre-eclampsia is also challenging, particularly as no intermediate complication exists between severe systolic hypertension (relatively common) and stroke (very rare in high-income health-care settings), and treatment paradox could mean that women are appropriately delivered on the basis of moderate deterioration in biochemical parameters before severe complications occur. The incidences of maternal and perinatal primary outcomes were higher than anticipated on the basis of previous studies, but this did not limit the interpretation of the analysis. Although we acknowledge that for women enrolled after 36 weeks' gestation, expectant management would only be for a maximum of 7 days, immediate planned delivery would still represent a change in clinical care from usual practice and the research uncertainty remained when we conceived the trial. The proportion of women enrolled at 36 weeks' gestation was similar, and even slightly lower, than that enrolled in a similar trial,^15^ and maternal benefit was shown even at this gestation.

We considered sources of possible bias for our trial. Selection bias was unlikely due to the randomisation process, which included robust allocation sequence concealment such that determining next allocation was not possible. Performance and detection bias were possible because it was not possible to mask participating clinicians or women, nor data collectors because timing of delivery was contained within maternity records where morbidity was recorded. Every primary maternal outcome was additionally signed off by each site principal investigator, and we used a primary neonatal outcome (independently recorded by the attending clinical team) to minimise bias where possible. There was minimal attrition in both groups. We have reported all prespecified secondary outcomes, interpreting them cautiously.

We did not adjust for multiplicity for the co-primary outcomes, which fundamentally adheres to the concept that a clinical trial is a focused scientific research question. The key outcomes for mothers and their infants need to be considered together. Furthermore, statistical adjustment for multiple comparisons invokes debate among methodologists, and there is no consensus.

Our finding of a reduction in maternal adverse outcomes is similar to that of a previous study,^15^ but that trial included only 352 women with late preterm pre-eclampsia and the reduction was not statistically significant. However, our trial found no difference in respiratory morbidity (as a secondary outcome) and much higher antenatal corticosteroid use (60%) compared with the previous trial,^15^ which reported increased respiratory distress syndrome in those with planned delivery, with lower corticosteroid use (8%) and a longer interval to delivery in the expectant management group that was probably related to inclusion of women with chronic or gestational hypertension. Systematic reviews of planned early delivery in women with pregnancy hypertension to date have been constrained by insufficient numbers to draw definitive conclusions for specific groups of women in whom the benefit and risk balance might differ (ie, those with late preterm pre-eclampsia),^16^, ^17^ but an individual patient data meta-analysis^18^ has suggested that some women in these groups could benefit from earlier delivery. Developing accurate validated prognostic tools to best identify those at highest risk remains challenging, and infant follow-up is useful to further evaluate the longer term outcomes^19^ with such strategies.

In women with late preterm pre-eclampsia, planned delivery is associated with improved maternal outcomes but more neonatal unit admissions for prematurity (although not respiratory or other morbidity, higher intensity of neonatal care, or duration of stay) compared with expectant management. Although UK guidance does not recommend routine admission for prematurity alone, individual clinicians might vary in their thresholds for neonatal unit admission. Additional prolongation of pregnancy by 5 days, as seen in the expectant management group, might move an infant out of a notional group where admission is dictated by a guideline (eg, based on a gestational age threshold) rather than by clinical need. Increased use of transitional care arrangements, where an infant stays with their mother but with enhanced surveillance and care in a postnatal setting, might be particularly beneficial in these infants and avoid unnecessary separation of the infant from their mother. For women with pre-eclampsia at this gestational age, prolongation of pregnancy might only be for a few days; more than half of these women require indicated delivery, potentially necessitating emergency management. Rates of vaginal delivery are similar to those reported from a large US study^20^ of women with early preterm pre-eclampsia, suggesting that these results can be extrapolated across similar high-income settings. The increase in spontaneous vaginal births with planned delivery could be judged an important advantage by women and clinicians, particularly for future pregnancies. Notably, there were no stillbirths or neonatal deaths in this setting, and one maternal death was probably related to comorbidities in association with pre-eclampsia rather than treatment allocation. The benefits and risks of planned delivery in women with late preterm pre-eclampsia might vary in low-resource health-care settings and require further evaluation, although the potential disadvantages of increased prematurity would need to be balanced against a much higher incidence of stillbirth in women with pre-eclampsia managed expectantly, such as that reported in a South African setting.^21^ Our findings relate to women with late preterm pre-eclampsia and should not be extrapolated to women with chronic or gestational hypertension, in whom the likelihood of developing maternal morbidity is lower.

In conclusion, our trial supports offering initiation of delivery in women with late preterm pre-eclampsia. The trade-off of lower maternal morbidity and severe hypertension against higher neonatal unit admissions, albeit without additional respiratory or other morbidity, should be discussed with women with late preterm pre-eclampsia to allow shared decision making on timing of delivery.

## Data sharing

The dataset will be available to appropriate academic parties on request from the Chief Investigator (LCC) in accordance with the data sharing policies of King's College London and the National Perinatal Epidemiology Unit Clinical Trials Unit, with input from the Co-investigator group where applicable.
