# Supplementary material for: Planned early delivery or expectant management for late preterm pre-eclampsia (PHOENIX): a randomised controlled trial
Source: Lancet. 2019 Sep 28;394(10204):1181–90. doi: 10.1016/S0140-6736(19)31963-4 (PMC6892281; doi:10.1016/S0140-6736(19)31963-4)
Supplement: Supplementary appendix [file mmc1.pdf]

THE LANCET

Supplementary appendix

This appendix formed part of the original submission and has been peer reviewed. We post it as supplied by the authors.

Supplement to: Chappell LC, Brocklehurst P, Green ME, et al. Planned early delivery or expectant management for late preterm pre-eclampsia (PHOENIX): a randomised controlled trial. *Lancet* 2019; published online Aug 28. http://dx.doi.org/10.1016/S0140-6736(19)31963-4.

Contents

Supplementary Table 1: Recruitment by centre 2

Supplementary Table 2: Additional maternal demographic and pregnancy characteristics at trial entry .. 4

Supplementary Table 3: Additional secondary maternal outcomes 8

Supplementary Table 4: Process outcomes: time between randomisation to initiation of delivery and delivery........ 11

Supplementary Table 5: Additional secondary perinatal outcomes (Intention To Treat analysis) 12

Supplementary Table 6: Secondary perinatal outcomes post randomisation (by Per Protocol analysis) .. 15

Supplementary Table 7: Additional secondary perinatal outcomes (by Per Protocol analysis)........ 17

Supplementary Table 8: Unexpected maternal and infant serious adverse events by allocation 20

Supplementary Figure 1: Forest plot for sub-group analysis (Per Protocol population) of primary perinatal outcome comparing Planned Delivery (PD) with Expectant Management (EM). p-values compare risk ratios across the different sub-groups of each factor........ 21

Supplementary Table 9: Sensitivity analysis for the primary outcome, excluding women (maternal outcome) and babies (perinatal outcome) in the planned delivery arm for whom initiation of delivery was greater than 96 hours after randomisation 22

Members of the PHOENIX Study Group........ 23

Supplementary Table 1: Recruitment by centre

Centre	Planned delivery (n=448)	Expectant management (n=451)
St Thomas' Hospital, London	40 (9%)	40 (9%)
Darent Valley Hospital	22 (5%)	20 (4%)
St Mary's Hospital, Manchester	28 (6%)	22 (5%)
Bradford Royal Infirmary	16 (4%)	12 (3%)
West Middlesex University NHS Trust	26 (5%)	31 (7%)
Nottingham City Hospital	11 (3%)	15 (3%)
Leeds Teaching Hospitals - St James'	18 (4%)	15 (3%)
Liverpool Women's	22 (5%)	24 (5%)
Queens Medical Centre	10 (2%)	13 (3%)
Royal Victoria Infirmary	18 (4%)	21 (5%)
James Cook University Hospital	18 (4%)	19 (4%)
Sunderland Royal Hospital	17 (4%)	21 (5%)
University College Hospital	10 (2%)	14 (3%)
Birmingham Women's Hospital	11 (3%)	9 (2%)
St George's Hospital	8 (2%)	7 (2%))
Royal Stoke University Hospital	7 (2%)	7 (2%)
Western Sussex Hospitals	7 (2%)	11 (2%)
Whittington Hospital	4 (1%)	3 (1%)
ABM University Hospitals, Wales	23 (5%)	22 (5%)
Birmingham City Hospital	8 (2%)	7 (2%)
Birmingham Heartlands Hospital	6 (1%)	1 (0%)
Warrington and Halton Hospitals	2 (0%)	3 (1%)
Chesterfield Royal Hospital	5 (1%)	8 (2%)
Royal United Hospital, Bath	4 (1%)	5 (1%)
Kingston Hospital NHS Trust	16 (4%)	11 (2%)
Leighton Hospital	6 (1%)	7 (2%)
Leicester Royal infirmary	8 (2%)	8 (2%)
Shrewsbury and Telford Hospital NHS Trust	2 (0%)	3 (1%)
Royal Preston Hospital	2 (0%)	3 (1%)
Northampton General	6 (1%)	7 (1%)
Gloucestershire Royal Hospital	0 (0%)	2 (0%)

St Michael's Hospital, Bristol	2 (0%)	5 (1%)
Royal London Hospital	5 (1%)	5 (1%)
Whipps Cross Hospital	9 (2%)	7 (2%)
New Cross Hospital, wolverhampton	4 (1%)	4 (1%)
Cambridge University Hospitals	3 (1%)	0 (0%)
Chelsea and Westminster Hospital	2 (0%)	5 (1%)
Royal Bolton Hospital	4 (1%)	2 (0%)
St Helier Hospital	2 (0%)	2 (0%)
University Hospital, Lewisham	0 (0%)	2 (0%)
Luton and Dunstable	1 (0%)	0 (0%)
Epsom Hospital	2 (0%)	0 (0%)
Queen Elizabeth Hospital, Greenwich	5 (1%)	1 (0%)
Queen's Hospital, Romford	2 (0%)	2 (0%)
Croydon University Hospital	23 (5%)	25 (6%)
Broomfield Hospital, Chelmsford	3 (1%)	0 (0%)

Supplementary Table 2: Additional maternal demographic and pregnancy characteristics at trial entry

	**Planned delivery (n = 448)**	**Expectant management (n = 451)**
**Median (IQR) gestational age at booking (weeks)**	10 (8 to 12)	10 (8 to 12)
**Deprivation Index quintile (England only)**		
1 (Least deprived)	41/425 (10%)	25/428 (6%)
2	53/425 (13%)	53/428 (12%)
3	64/425 (15%)	72/428 (17%)
4	106/425 (25%)	118/428 (28%)
5 (Most deprived)	161/425 (38%)	160/428 (37%)
Centre in Wales (quintiles not available)	23	22
**Parity (previous pregnancies ≥24 weeks' gestation)***		
0	254 (57%)	260 (58%)
1	104 (23%)	103 (23%)
2	49 (11%)	52 (12%)
>2	41 (9%)	36 (8%)
**Mode of previous deliveries**		
Total number of previous deliveries	368	335
Spontaneous vaginal delivery	241/368 (66%)	201/335 (60%)
Assisted vaginal delivery	20/368 (5%)	24/335 (7%)
Caesarean section	102/368 (28%)	110/335 (33%)
Unknown	5/368 (1%)	0/335 (0%)
**Previous pregnancies <24 weeks' gestation**		
0	295 (66%)	309 (69%)
1	96 (21%)	87 (19%)
2	28 (6%)	33 (7%)
>2	29 (7%)	22 (5%)
**Body mass index (kg/m^2)**		
<18.5	4 (1%)	4 (1%)
18.5-24.9	126 (28%)	114 (25%)
25.0-29.9	120 (27%)	146 (32%)
30.0-39.9	161 (36%)	153 (34%)
≥40·0	37 (8%)	34 (8%)

**First trimester Pregnancy-associated plasma protein-A Multiple of the Median**		
Number recorded	159 (36%)	172 (39%)
Median (IQR)	0·80 (0·51 to 1·27)	0·85 (0·51 to 1·35)
**Second trimester uterine artery Doppler mean pulsatility index**		
Number recorded	89 (20%)	91 (20%)
Median (IQR)	1·20 (0·94 to 1·49)	1·09 (0·88 to 1·49)
**Parameters related to diagnosis of pre-eclampsia**		
**Highest blood pressure reading that led to diagnosis (mmHg)**		
If based on single diastolic blood pressure ≥110mmHg: n (%)	53 (12%)	64 (14%)
Mean (SD) systolic blood pressure	163·8 (16·76)	166·1 (15·07)
Mean (SD) diastolic blood pressure	114·2 (6·30)	114·1 (5·24)
If based on 2 diastolic blood pressure readings ≥90mmHg, mean of 2 readings: n (%)	395 (88%)	387 (86%)
Mean (SD) systolic blood pressure	149·7 (9·99)	150·0 (10·19)
Mean (SD) diastolic blood pressure	96·7 (4·37)	96·5 (4·06)
All participants: n (%)	448 (100%)	451 (100%)
Mean (SD) systolic blood pressure	151·4 (11·88)	152·3 (12·36)
Mean (SD) diastolic blood pressure	98·8 (7·30)	99·0 (7·47)
**Additional parameters leading to diagnosis of pre-eclampsia (non-exclusive)**		
Urinary protein-creatinine ratio ≥30 (mg/mmol): n (%)	405 (90%)	407 (90%)
Median (IQR) (if ≥ 30 mg/mmol)	92 (50 to 188)	87 (47 to 187)
24 hr urinary protein excretion ≥300 (mg/24hrs): n (%)	18 (4%)	21 (5%)
Median (IQR) (if ≥300 mg/24hrs)	550 (400 to 1000)	900 (600 to 50000)
Creatinine >90 µmol/L: n (%)	14 (3%)	11 (2%)
Median (IQR) (if >90 µmol/L)	100 (94 to 110)	99 (91 to 111)
Alanine or aspartate transaminase >70 IU/L: n (%)	19 (4%)	15 (3%)
Median (IQR) (if >70 IU/L)	107 (84 to 211)	105 (83 to 317)
Platelet count <150 x 10^9/L: n (%)	44 (10%)	43 (10%)
Median (IQR) (if <150 x 10^9/L)	130 (108 to 140)	131 (118 to 144)
Estimated fetal weight <10th centile on ultrasound	61 (14%)	66 (15%)
**Antihypertensive medication at study entry**	359 (80%)	374 (83%)

One oral agent	240/359 (67%)	241/374 (64%)
Two or more oral agents	117/359 (33%)	132/374 (35%)
One intravenous agent	2/359 (1%)	5/374 (1%)
**Aspirin prescribed during pregnancy**	170 (38%)	189 (42%)
Median (IQR) gestational age aspirin first prescribed (weeks)	13 (11 to 16)	12 (11 to 16)
**Low molecular weight heparin prescribed during pregnancy**	125 (28%)	117 (26%)
Median (IQR) Gestational age heparin first prescribed (weeks)	33 (31 to 34)	34 (31 to 35)
**Most recent proteinuria reading**		
Urinary protein-creatinine ratio recorded: n (%)	434 (97%)	441 (98%)
Median (IQR) (mg/mmol)	83 (42 to 186)	80 (42 to 172)
24 hr urinary protein excretion recorded: n (%)	15 (3%)	14 (3%)
Median (IQR) (mg/24hrs)	78 (31 to 550)	107 (31 to 460)
**Most recent lab parameters prior to study entry**		
Median (IQR) haemoglobin (g/L)	116 (108 to 124)	117 (108 to 124)
Median (IQR) platelet count ($x10^9$/L)	213 (174 to 255)	210 (174 to 251)
Median (IQR) creatinine (μmol/L)	59 (50 to 68)	59 (51 to 67)
Median (IQR) alanine aminotransferase (U/L)	15 (10 to 22)	15 (10 to 22)
n (%)	424 (95%)	422 (94%)
Median (IQR) aspartate aminotransferase (U/L)	20 (15 to 32)	20 (15 to 24)
n (%)	51 (11%)	59 (13%)
Median (IQR) Placental growth factor (pg/ml)	16 (12 to 43)	12 (12 to 22)
n (%)	33 (7%)	28 (6%)
Median (IQR) uric acid (μmol/L)	366 (303 to 416)	357 (300 to 424)
n (%)	166 (37%)	165 (37%)
**Bishop score assessed at study entry**	9 (2%)	6 (1%)
<2	2/9 (22%)	2/6 (33%)
2-6	7/9 (78%)	4/6 (67%)
**Fetal growth scan in last two weeks**	366 (82%)	375 (83%)
Suspected fetal growth restriction	79/366 (22%)	85/375 (23%)
Indicator of fetal growth restriction (non-exclusive)		
Abdominal circumference <10th centile	23 (5%)	32 (7%)
Estimated fetal weight <10th centile	67 (15%)	73 (16%)

Umbilical artery pulsatility index >95th centile	8 (2%)	13 (3%)
Absent or reversed umbilical artery end diastolic flow	2 (0%)	5 (1%)
Amniotic fluid index <5th centile	4 (1%)	7 (2%)
**In-patient at time of trial entry**	362 (81%)	371 (82%)

Data are n (%) unless shown otherwise. n/N (%) indicates that the denominator only includes participants with a relevant measurement for that variable.

Supplementary Table 3: Additional secondary maternal outcomes

	Planned delivery (n=448)	Expectant management (n=451)
**Components by category (non-exclusive)**		
Maternal death	0 (0%)	1 (0%)
**Central nervous system**		
Eclampsia	3 (1%)	4 (1%)
Glasgow coma score <13	0 (0%)	0 (0%)
Stroke or reversible ischaemic neurological deficit	0 (0%)	0 (0%)
Transient ischaemic attack	0 (0%)	0 (0%)
Cortical blindness or retinal detachment	0 (0%)	0 (0%)
Posterior reversible encephalopathy	0 (0%)	0 (0%)
**Cardiorespiratory**		
Positive inotropic support	0 (0%)	1 (0%)
Infusion of a third parenteral antihypertensive drug	2 (0%)	0 (0%)
Myocardial ischaemia or infarction	1 (0%)	0 (0%)
SpO_2 <90%	2 (0%)	3 (1%)
≥50% FiO_2 for >1 hr	1 (0%)	0 (0·0)
Intubation (other than for caesarean section)	2 (0%)	0 (0%)
Pulmonary oedema	1 (0%)	2 (0%)
**Haematological**		
Transfusion of any blood product	20 (5%)	23 (5%)
Platelet count <50×10^9 per L, with no transfusion	2 (0%)	4 (1%)
**Hepatic**		
Hepatic dysfunction	44 (10%)	63 (14%)
Hepatic haematoma or rupture	0 (0%)	0 (0%)
**Renal**		
Acute renal insufficiency (creatinine >150 µmol/L; no pre-existing renal disease)	3 (1%)	4 (1%)
Acute renal failure (creatinine >200 µmol/L; pre-existing renal disease)	0 (0%)	0 (0%)
Dialysis	0 (0%)	0 (0%)
**Other**		

Placental abruption	4 (1%)	4 (1%)
**Systolic blood pressure ≥160 mmHg post randomisation**	267 (60%)	313 (70%)
Systolic blood pressure ≥160mmHg (randomisation to delivery)	203 (45%)	261 (58%)
Systolic blood pressure ≥160mmHg (delivery to post-delivery discharge)	172 (39%)	173 (39%)
**Highest BP recorded: randomisation to delivery**		
Mean (SD) systolic blood pressure (mmHg)	159·4 (17%)	164·4 (17%)
Mean (SD) diastolic blood pressure (mmHg)	95·0 (11%)	97·7 (12%)
**Highest BP recorded: delivery to post-delivery discharge**		
Mean (SD) systolic blood pressure (mmHg)	155·8 (16%)	156·6 (16%)
Mean (SD) diastolic blood pressure (mmHg)	91·5 (12%)	92·5 (12%)
**Antihypertensive medication: randomisation to delivery**	381 (85%)	405 (90%)
One oral agent	233/381 (61%)	185/405 (46%)
Two or more oral agents	147/381 (39%)	218/405 (54%)
One intravenous agent	17/381 (5%)	37/405 (9%)
Two or more intravenous agents	3/381 (1%)	4/405 (1%)
**Anti-hypertensive drugs administered (non-exclusive)**		
Hydralazine	10/381 (3%)	25/405 (6%)
Labetalol	328/381 (86%)	353/405 (87%)
Methyldopa	39/381 (10%)	61/405 (15%)
Nifedipine	162/381 (43%)	222/405 (55%)
Amlodipine	5/381 (1%)	5/405 (1%)
Atenolol	2/381 (1%)	3/405 (1%)
Diltiazem	0/381 (0%)	0/405 (0%)
Doxazosin	2/381 (1%)	6/405 (2%)
Ketaserin	0/381 (0%)	0/405 (0%)
Propranolol	0/381 (0%)	1/405 (0%)
Verapamil	0/381 (0%)	0/405 (0%)
Other	2/381 (1%)	3/405 (1%)
Progression to HELLP syndrome	7 (2%)	10 (2%)
Magnesium sulfate: randomisation to delivery	31 (7%)	69 (15%)
Low molecular weight heparin: randomisation to delivery	157 (35%)	208 (46%)
Steroids for fetal lung maturation	291 (65%)	248 (55%)

Number of doses of steroids		
1	12/291 (4%)	14/248 (6%)
2	277/291 (95%)	229/248 (92%)
3	0/291 (0%)	0/248 (0%)
≥4	2/291 (1%)	5/248 (2%)
**Suspected fetal growth restriction post randomisation**	13 (3%)	41 (9%)
**Indicator of fetal growth restriction (non-exclusive)**		
Abdominal circumference <10th centile	6	15
Estimated fetal weight <10th centile	8	28
Umbilical artery pulsatility index >95th centile	3	13
Absent or reversed umbilical artery end diastolic flow	2	3
Amniotic fluid index <5th centile	2	7
**Onset of labour**		
Induced	304 (68%)	275 (61%)
**Method of induction if induced (non-exclusive)**		
Prostaglandin gel/pessary	275/304 (91%)	238/275 (87%)
Foley catheter	13/304 (4%)	7/275 (3%)
Artificial rupture of membranes	133/304 (44%)	120/275 (44%)
Syntocinon	99/304 (33%)	90/275 (33%)
Other	1/304 (0%)	3/275 (1%)
**Estimated amount of blood loss at delivery (mls)**		
Mean (SD)	559·0 (583·1)	557·9 (454·8)
Median (IQR)	406 (300 to 600)	400 (300 to 650)
**Confirmed maternal sepsis (positive blood or urine cultures) between randomisation and hospital discharge**	2 (0%)	6 (1%)
Blood culture	1/2 (50%)	5/6 (83%)
Urine culture	1/2 (50%)	1/6 (17%)

Data are n (%) unless shown otherwise· n/N (%) indicates that the denominator only includes participants with a relevant measurement for that variable· HELLP: haemolysis, elevated liver enzymes, low platelets syndrome.

Supplementary Table 4: Process outcomes: time between randomisation to initiation of delivery and delivery

	Planned delivery (n = 448)	Expectant management (n = 451)
**Time between randomisation and initiation of delivery (days)**		
**Overall**		
n	447	450
Median (IQR)	1 (1 to 2)	5 (3 to 8)
**By gestational age at randomisation**		
**34^{+0} to 34^{+6}**		
n (%)	131 (29%)	135 (30%)
Median (IQR)	1 (1 to 2)	6 (2 to 11)
**35^{+0} to 35^{+6}**		
n (%)	136 (30%)	131 (29%)
Median (IQR)	1 (1 to 2)	6 (3 to 10)
**36^{+0} to 36^{+6}**		
n (%)	180 (40%)	184 (41%)
Median (IQR)	1 (1 to 2)	4 (2 to 6)
**Time between randomisation and delivery (days)**		
**Overall**		
n	447	451
Median (IQR)	2 (1 to 4)	6 (3 to 9)
**By gestational age at randomisation**		
**34^{+0} to 34^{+6}**		
n (%)	131 (29%)	135 (30%)
Median (IQR)	3 (1 to 4)	7 (3 to 12)
**35^{+0} to 35^{+6}**		
n (%)	136 (30%)	132 (29%)
Median (IQR)	2 (1 to 3)	7 (4 to 11)
**36^{+0} to 36^{+6}**		
n (%)	180 (40%)	184 (41%)
Median (IQR)	2 (2 to 4)	5 (4 to 7)

Supplementary Table 5: Additional secondary perinatal outcomes (Intention To Treat analysis)

	**Planned delivery (n = 471)**	**Expectant management (n = 475)**
**Mode of delivery**		
Spontaneous vaginal	169 (36%)	139 (29%)
Spontaneous vaginal (cephalic)	165 (35%)	138 (29%)
Spontaneous vaginal (breech)	4 (1%)	1 (0%)
Assisted vaginal (cephalic)	40 (9%)	47 (10%)
Assisted vaginal – vacuum	13 (3%)	19 (4%)
Assisted vaginal – forceps	27 (6%)	28 (6%)
Caesarean section	260 (55%)	289 (61%)
Pre-labour caesarean section	153 (33%)	168 (35%)
Emergency caesarean section	107 (23%)	121 (26%)
**Indication for assisted vaginal delivery (non-exclusive)**		
Maternal comorbidity or disease	1/40 (3%)	3/47 (6%)
Failure to progress in second stage	16/40 (40%)	17/47 (36%)
Suspected fetal distress	28/40 (70%)	30/47 (64%)
**Indication for caesarean delivery (non-exclusive)**		
Maternal comorbidity or disease	40/260 (15%)	78/289 (27%)
Previous caesarean delivery/uterine surgery	57/260 (22%)	57/289 (20%)
Failure to progress in first stage	39/260 (15%)	46/289 (16%)
Failure to progress in second stage	2/260 (1%)	1/289 (0%)
Suspected fetal distress	83/260 (32%)	101/289 (35%)
Failed instrumental delivery	3/260 (1%)	1/289 (0%)
Fetal presentation not cephalic	42/260 (16%)	31/289 (11%)
Twins	6/260 (2%)	9/289 (3%)
Maternal request	7/260 (3%)	1/289 (0%)
**Gestational age at delivery (days)**		
<37 weeks	387 (83%)	261 (55%)
**Baby sex**		
Boy	240 (51%)	233 (49%)
Girl	229 (49%)	242 (51%)
**Umbilical artery pH <7·05**<br>**Umbilical arterial pH collected**	6/281 (2%)<br>281 (60%)	7/266 (3%)<br>266 (56%)

**Principal recorded indication for neonatal unit admission**		
**Number of babies admitted to the neonatal unit***	196	159
**Preterm**		
Prematurity	83 (42%)	40 (25%)
**Cardiorespiratory**		
Respiratory disease	47 (24%)	41 (26%)
Cardiovascular disease	0 (0%)	1 (1%)
Failed oximetry testing	0 (0%)	1 (1%)
**Gastric, Hepatic, Metabolic**		
Gastrointestinal tract disease	0 (0%)	0 (0%)
Jaundice	12 (6%)	11 (7%)
Hypoglycaemia	21 (11%)	31 (20%)
Other metabolic disease	0 (0%)	0 (0%)
**Neurological**		
Convulsions	1 (1%)	0 (0%)
Hypoxic ischaemic encephalopathy	0 (0%)	0 (0%)
Poor condition at birth	2 (1%)	3 (2%)
Neonatal abstinence syndrome	0 (0%)	0 (0%)
Other neurological disease	0 (0%)	0 (0%)
**Infection**		
Infection suspected/confirmed	9 (5%)	12 (8%)
**Size, weight, feeding**		
Intrauterine growth restriction/ Small for gestational age infant	8 (4%)	10 (6%)
Poor feeding or weight loss	4 (2%)	2 (1%)
**Anomaly or trauma**		
Congenital anomaly suspected/ confirmed	2 (1%)	0 (0%)
Birth trauma/ injury	0 (0%)	0 (0%)
**Relating to carer**		
Social issues/Foster care	0 (0%)	0 (0%)
Maternal admission/emergency	1 (1%)	2 (1%)
**Specialist care**		
Surgery	0 (0%)	0 (0%)
Palliative care	0 (0%)	0 (0%)

**Monitoring or investigation**		
Monitoring	4 (2%)	5 (3%)
Exclusively for specific investigation	0 (0%)	0 (0%)
Continuing care	2 (1%)	0 (0%)
**Need for respiratory support**	45 (10%)	48 (10%)
**Type of respiratory support needed (non-exclusive)**		
Endotracheal ventilation	10/45 (22%)	14/48 (29%)
Continuous positive airway pressure	36/45 (80%)	37/48 (77%)
High flow oxygen	12/45 (27%)	17/48 (35%)
**Cerebral ultrasound scan performed**	18 (4%)	24 (5%)
**Abnormalities found**	2/18 (11%)	1/24 (4%)
Intraventricular haemorrhage (IVH) Grade I-II	1/2 (50%)	0/1 (0%)
Intraventricular haemorrhage (IVH) causing ventricular distension	1/2 (50%)	0/1 (0%)
Other	0/2 (0%)	1/1 (100%)
**Sepsis confirmed**	3 (1%)	2 (0%)
Positive blood cultures	3/3 (100%)	1/2 (50%)
Cerebrospinal fluid cultures	0/3 (0%)	1/2 (50%)
**Necrotising enterocolitis confirmed (Bell's stage 2 or 3)**	0 (0%)	0 (0%)
**Seizures**	0 (0%)	0 (0%)
**Diagnosed Encephalopathy**	0 (0%)	0 (0%)
**Diagnosed hypoglycaemia (blood glucose <2·6 mmol/L on ≥2 consecutive occasions)**	80 (17%)	72 (15%)
Intravenous dextrose required	32/80 (40%)	32/72 (44%)
Tube feeding required	41/80 (51%)	45/72 (63%)
**Method of infant feeding 24 hrs prior to discharge**		
Exclusive breast-feeding	112 (24%)	139 (30%)
Mixed feeding	174 (38%)	161 (34%)
Exclusive formula feeding	176 (38%)	168 (36%)

Data are n (%) unless shown otherwise· n/N (%) indicates that the denominator only includes participants with a relevant measurement for that variable.

*Number of babies admitted to the neonatal unit (denominator for the principal recorded indications).

Supplementary Table 6: Secondary perinatal outcomes post randomisation (by Per Protocol analysis)

	**Planned delivery (n = 342)**	**Expectant management (n = 470)**	**Adjusted effect measure* (95% CI)**
Stillbirth	0 (0%)	0 (0%)	-
Neonatal deaths within 7 days of delivery	0 (0%)	0 (0%)	-
Neonatal death before discharge	0 (0%)	0 (0%)	-
Median (IQR) gestational age at delivery (days)	252 (246 to 257)	258 (251 to 260)	-4·0 (-4·6, -3·4)
**Mode of delivery**			
Spontaneous vaginal	127 (37%)	138 (29%)	1·21 (1·01, 1·44)
Assisted vaginal	34 (10%)	46 (10%)	0·97 (0·64, 1·46)
Caesarean section	181 (53%)	286 (61%)	0·90 (0·81, 1·00)
Median (IQR) birth weight (g)	2400 (2060 to 2780)	2483 (2150 to 2912)	-109 (-167, -51)
Median (IQR) birth weight centile†	34 (17 to 61)	30 (13 to 61)	2·5 (-2·3, 7·4)
Birth weight <10th centile	53 (16%)	95 (20%)	0·77 (0·56, 1·05)
Birth weight <3rd centile	13 (4%)	27 (6%)	0·69 (0·36, 1·33)
Median (IQR) Apgar score at 5 minutes post birth	10 (9 to 10)	10 (9 to 10)	-
Median (IQR) umbilical arterial pH<br>Umbilical arterial pH collected	7·26 (7·19 to 7·30)<br>201 (59%)	7·25 (7·20 to 7·30)<br>265 (56%)	0·00 (-0·01, 0·02)
Number of infants admitted to neonatal unit	155 (45%)	155 (33%)	1·40 (1·18, 1·66)
**Principal recorded indication for neonatal unit admission**			
Prematurity	66/155 (43%)	38/155 (25%)	
Respiratory disease	34/155 (22%)	39/155 (25%)	
Hypoglycaemia	19/155 (12%)	31/155 (20%)	
Jaundice	10/155 (7%)	11/155 (7%)	
Infection suspected/confirmed	7/155 (5%)	12/155 (8%)	
Intrauterine growth restriction/ Small for gestational age infant	7/155 (5%)	10/155 (7%)	
Other	12/155 (8%)	14/155 (9%)	
Need for respiratory support	32 (9%)	46 (10%)	0·98 (0·55, 1·77)
Need for supplementary oxygen prior to discharge	47 (14%)	47 (10%)	1·41 (0·97, 2·07)
Median (IQR) supplemental oxygen required	1 (1 to 2)	2 (1 to 3)	

Cerebral ultrasound abnormalities found/ number tested	0/12 (0%)	1/20 (5%)	
Sepsis confirmed	1 (0%)	2 (0%)	
Necrotising enterocolitis	0 (0%)	0 (0%)	
Seizures	0 (0%)	0 (0%)	
Encephalopathy	0 (0%)	0 (0%)	
Hypoglycaemia during neonatal unit admission	61 (18%)	71 (15%)	
Exclusive breast-feeding 24 hrs prior to discharge	84 (25%)	139 (30%)	
**Total neonatal unit stay**			
Median (IQR) days in neonatal unit<br>Number (%) admitted for at least one day	6 (2 to 10)<br>147 (43%)	6 (3 to 12)<br>149 (32%)	-0·4 (-2·07, 1·21)
**Category of care during neonatal unit stay (separation of baby from mother)**			
Median (IQR) days in intensive care<br>Number (%) admitted	2 (1 to 3)<br>22 (6%)	3 (1 to 4)<br>19 (4%)	-1·4 (-27, 25)
Median (IQR) days in high dependency care<br>Number (%) admitted	1 (1 to 3)<br>39 (11%)	2 (1 to 5)<br>32 (7%)	-0·6 (-3·3, 2·2)
Median (IQR) days in special care<br>Number (%) admitted	5 (2 to 10)<br>135 (40%)	6 (2 to 11)<br>139 (30%)	0 (-1·7, 1·7)
**Category of care during other postnatal stay (baby alongside mother)**			
Median (IQR) days in transitional care<br>Number (%) admitted	6 (2 to 8)<br>26 (8%)	5 (4 to 6)<br>16 (3%)	-
Median (IQR) days in postnatal care<br>Number (%) admitted	3 (2 to 5)<br>249 (73%)	3 (2 to 4)<br>382 (82%)	0·25 (-0·06, 0·56)

Data are n (%) unless otherwise stated. Effect measures are risk ratios for categorical variables (risk in planned delivery group/ risk in expectant management group) and median differences for continuous variables (median in planned delivery group - median in expectant management group). CI: confidence intervals; IQR: interquartile range· n/N (%) indicates that the denominator only includes participants with a relevant measurement for that variable.

*Adjusted for centre, singleton/twin pregnancies, severity of hypertension in 48 hours prior to enrolment, parity, previous caesarean section and gestational age at randomisation.

† Birth weight centile calculated using the Stata add-in function zanthro using the British 1990 Growth Reference (reanalysed 2009).

Supplementary Table 7: Additional secondary perinatal outcomes (by Per Protocol analysis)

	Planned delivery (n = 342)	Expectant management (n = 470)
**Mode of delivery**		
Spontaneous vaginal	127 (37%)	138 (29%)
Spontaneous vaginal (cephalic)	125 (37%)	137 (29%)
Spontaneous vaginal (breech)	2 (1%)	1 (0%)
Assisted vaginal (cephalic)	34 (10%)	46 (10%)
Assisted vaginal – vacuum	12 (4%)	19 (4%)
Assisted vaginal – forceps	22 (6%)	27 (6%)
Caesarean section	181 (53%)	286 (61%)
Pre-labour caesarean section	92 (27%)	167 (36%)
Emergency caesarean section	89 (26%)	119 (25%)
**Indication for assisted vaginal delivery (non-exclusive)**		
Maternal comorbidity or disease	1/34 (3%)	3/46 (7%)
Failure to progress in second stage	13/34 (38%)	17/46 (37%)
Suspected fetal distress	25/34 (74%)	29/46 (63%)
**Indication for caesarean delivery (non-exclusive)**		
Maternal comorbidity or disease	25/181 (14%)	75/286 (26%)
Previous caesarean delivery/uterine surgery	30/181 (17%)	55/286 (19%)
Failure to progress in first stage	31/181 (17%)	46/286 (16%)
Failure to progress in second stage	2/181 (1%)	1/286 (0%)
Suspected fetal distress	66/181 (37%)	101/286 (35%)
Failed instrumental delivery	3/181 (2%)	1/286 (0%)
Fetal presentation not cephalic	33/181 (18%)	31/286 (11%)
Twins	2/181 (1%)	9/286 (3%)
Maternal request	5/181 (3%)	1/286 (0%)
**Gestational age at delivery (days)**		
<37 weeks	286 (84%)	256 (55%)
**Baby sex**		
Boy	176 (52%)	229 (49%)
Girl	166 (49%)	241 (51%)
**Umbilical artery pH <7·05**	5/201 (3%)	7/265 (3%)

**Umbilical arterial pH collected**	201 (59%)	265 (57%)
**Principal recorded indication for neonatal unit admission**		
**Number of babies admitted to the neonatal unit***	155	155
**Preterm**		
Prematurity	66 (43%)	38 (25%)
**Cardiorespiratory**		
Respiratory disease	34 (22%)	39 (25%)
Cardiovascular disease	0 (0%)	1 (1%)
Failed oximetry testing	0 (0%)	1 (1%)
**Gastric, Hepatic, Metabolic**		
Gastrointestinal tract disease	0 (0%)	0 (0%)
Jaundice	10 (7%)	11 (7%)
Hypoglycaemia	19 (12%)	31 (20%)
Other metabolic disease	0 (0%)	0 (0%)
**Neurological**		
Convulsions	1 (1%)	0 (0%)
Hypoxic ischaemic encephalopathy	0 (0%)	0 (0%)
Poor condition at birth	2 (1%)	3 (2%)
Neonatal abstinence syndrome	0 (0%)	0 (0%)
Other neurological disease	0 (0%)	0 (0%)
**Infection**		
Infection suspected/confirmed	7 (5%)	12 (8%)
**Size, weight, feeding**		
Intrauterine growth restriction/ Small for gestational age infant	7 (5%)	10 (7%)
Poor feeding or weight loss	3 (2%)	2 (1%)
**Anomaly or trauma**		
Congenital anomaly suspected/ confirmed	0 (0%)	0 (0%)
Birth trauma/ injury	0 (0%)	0 (0%)
**Relating to carer**		
Social issues/Foster care	0 (0%)	0 (0%)
Maternal admission/emergency	0 (0%)	2 (1%)
**Specialist care**		
Surgery	0 (0%)	0 (0·0)

Palliative care	0 (0%)	0 (0·0)
**Monitoring or investigation**		
Monitoring	4 (3%)	5 (3%)
Exclusively for a specific investigation	0 (0%)	0 (0%)
Continuing care	2 (1%)	0 (0%)
**Need for respiratory support**	32 (9%)	46 (10%)
**Type of respiratory support needed (non-exclusive)**		
Endotracheal ventilation	6/32 (19%)	14/46 (30%)
Continuous positive airway pressure	27/32 (84%)	35/46 (76%)
High flow oxygen	8/32 (25%)	17/46 (37%)
**Cerebral ultrasound scan performed**	12 (4%)	20 (4%)
**Abnormalities found**	0/12 (0%)	1/20 (5%)
Intraventricular haemorrhage (IVH) Grade I-II	0/0 (0%)	0/1 (0%)
Intraventricular haemorrhage (IVH) causing ventricular distension	0/0 (0%)	0/1 (0%)
Other	0/0 (0%)	1/1 (100%)
**Sepsis confirmed**	1 (0%)	2 (0%)
Positive blood cultures	1/1 (100%)	1/2 (50%)
Cerebrospinal fluid cultures	0/1 (0%)	1/2 (50%)
**Diagnosed Encephalopathy**	0 (0%)	0 (0%)
**Diagnosed hypoglycaemia (blood glucose <2·6 mmol/L on ≥2 consecutive occasions)**	61 (18%)	71 (15%)
Intravenous dextrose required	28/61 (46%)	31/71 (44%)
Tube feeding required	33/61 (54%)	44/71 (62%)
**Method of infant feeding 24 hrs prior to discharge**		
Exclusive breast-feeding	84 (25%)	139 (30%)
Mixed feeding	124 (37%)	159 (34%)
Exclusive formula feeding	128 (38%)	167 (36%)

Data are n (%) unless shown otherwise· n/N (%) indicates that the denominator only includes participants with a relevant measurement for that variable.

*Number of babies admitted to the neonatal unit (denominator for the principal recorded indications).

Supplementary Table 8: Unexpected maternal and infant serious adverse events by allocation

	**Planned delivery**	**Expectant management**
**Serious adverse events (SAEs)**	9	12
**Severity**		
Mild	4	3
Moderate	2	2
Severe	3	7
**Causality**		
Not related	7	9
Possibly	2	2
Probably	0	1
**Action taken**		
Intervention stopped prior to the event started	2	5
None	7	7
**Outcome**		
Fatal	0	1
Not resolved	0	3
Resolved	6	7
Resolved with sequelae	1	0
Resolving	2	1
**System Organ Class**		
Cardiac disorders	3	3
Gastrointestinal disorders	0	1
Infections and infestations	1	0
Neoplasms benign, malignant and unspecified	0	1
Pregnancy, puerperium and perinatal conditions	4	5
Renal and urinary disorders	0	1
Respiratory, thoracic and mediastinal disorders	0	1
Vascular disorders	1	0

Supplementary Figure 1: Forest plot for sub-group analysis (Per Protocol population) of primary perinatal outcome comparing Planned Delivery (PD) with Expectant Management (EM). p-values compare risk ratios across the different sub-groups of each factor.

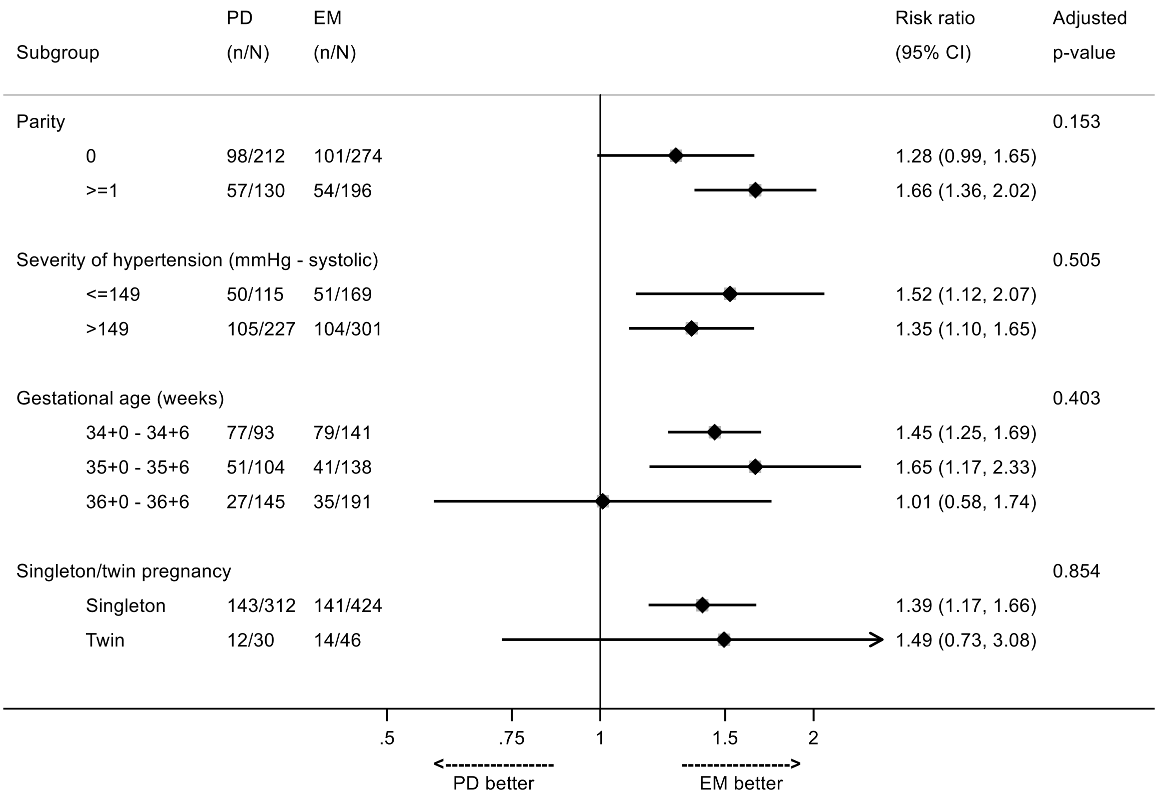

Supplementary Table 9: Sensitivity analysis for the primary outcome, excluding women (maternal outcome) and babies (perinatal outcome) in the planned delivery arm for whom initiation of delivery was greater than 96 hours after randomisation

	**Planned delivery**	**Expectant management**	**Risk ratio (95% CI) p-value**	**Adjusted risk ratio (95% CI) p-value**
Composite of maternal morbidity and/or recorded systolic BP ≥160 mmHg post randomisation	274/429 (64%)	338/451 (75%)	0·85 (0·78, 0·93) 0.0003	0·85 (0·78, 0·93) 0.0003
Composite of perinatal deaths and NNU admissions up to infant hospital discharge	184/450 (41%)	159/475 (34%)	1·23 (1·03, 1·46) 0·0205	1·24 (1·06, 1·44) 0·0075

Data are n (%).

*Adjusted for centre, singleton/twin pregnancies, severity of hypertension in 48 hours prior to enrolment, parity, previous caesarean section and gestational age at randomisation.

Members of the PHOENIX Study Group

Umber Agarwal, Irshad Ahmed, Bini Ajay, Zarko Alfirevic, Rita Arya, Gabrielle Bambridge, Jacqueline Bamfo, Sambita Basak, Ursula Bowler, Helen Cameron, David Churchill, Janet Cresswell, Fiona Crosfill, Mark Denbow, Madhuchanda Dey, Caroline Everden, Jo Ficquet, Katarzyna Gajewska-Knapik, Ramesh Ganapathy, Angela Garrett, Joanna Girling, Adam Gornall, Kate Harding, Eleanor Hendy, Richard Howard, Mark James, Antoinette Johnson, Michelle Kemp, Asma Khalil, Rehan Khan, Rahila Khan, Ellen Knox, Lavinia Margarit, Philippa Marsden, Karen McIntyre, Jenny Myers, Justine Nugent, Sanjay Rao, Zoey Robinson, Stephen Robson, Pauline Rushby, Laura Scholz, Mohamed Shahin, Bhavna Sharma, Nigel Simpson, Natasha Singh, Jenie Sparkes, Sophia Stone, Seni Subair, Bee Tan, Vidya Thakur, Sujatha Thamban, Jim Thornton, Sue Tohill, Elly Tsoi, Derek Tuffnell, Mark Waterstone, Jason Waugh, Cornelia Wiesender, Pensee Wu
